# Unraveling the Genetic Code: The Legacy of Har Gobind Khorana (January 9, 1922, to November 9, 2011)

**DOI:** 10.7759/cureus.60048

**Published:** 2024-05-10

**Authors:** Kamna Bansal

**Affiliations:** 1 Family and Community Medicine, Baylor College of Medicine, Houston, USA

**Keywords:** innovations, biography, nobel prize, genetic code, khorana

## Abstract

Dr. Har Gobind Khorana was an innovative chemist and Nobel laureate whose groundbreaking research laid the foundation for our understanding of the genetic code and revolutionized the field of molecular biology. Born in 1922 in Punjab, India, his journey from a small village to academic greatness showcases his intellect and determination. Despite economic challenges, Khorana pursued education, earning scholarships for studies abroad. His scientific journey began with a Ph.D. in organic chemistry from the University of Liverpool, leading to postdoctoral research in Switzerland and the UK. Notably, Khorana's work at the University of Wisconsin-Madison earned him the Nobel Prize in 1968 for deciphering the genetic code. His legacy includes synthesizing coenzyme A, deciphering the genetic code, and creating artificial genes. Honored globally, he received accolades such as the Padma Vibhushan and was commemorated on a stamp. Beyond science, Khorana's humility and mentorship left a lasting impact. His life inspires others to keep learning and discovering new things about the world, motivating them to ask questions and unravel the mysteries of the universe.

## Introduction and background

Dr. Har Gobind Khorana, a pioneering biochemist and Nobel laureate, stands as a towering figure in the annals of scientific history. Born on January 9, 1922, under British colonial rule, Khorana's journey from a small village to the revered halls of academia represents an accomplishment of intellect and determination over hardship. Throughout his renowned career, Khorana's groundbreaking research laid the foundation for our understanding of the genetic code and modernized the field of molecular biology.

Early life and education

Khorana hailed from Raipur, a tiny village located in Punjab, India (now part of Pakistan). His father, an agricultural taxation clerk, held firm convictions regarding the importance of education [1]. Despite facing economic hardships and inadequate resources, Khorana not only finished high school but also pursued and completed both his bachelor’s and master’s degrees at Punjab University in Lahore. His academic ability earned him scholarships that enabled him to pursue higher education abroad [1,2].

In 1945, under a Government of India Fellowship, he moved to England to pursue a Ph.D. in organic chemistry at the University of Liverpool. Originally intending to explore the study of insecticides and fungicides, Khorana's academic path took an unexpected turn due to limited availability of space. Consequently, he was redirected to pursue studies in organic chemistry under the guidance of Roger J.S. Beer. He completed his Ph.D. while working on the chemistry of melanins [1,2].

## Review

Scientific journey 

Following the completion of his Ph.D. in 1948, Khorana started his incredible scientific journey of six decades that would span across continents and reshape our understanding of genetics. He did his post-doctoral research in Swiss Federal Institute of Technology (ETH) in Zurich. He worked on alkaloid chemistry there with Vladimir Prelog. His stay there was short-lived, only 11 months, as he did not receive any monetary support and had to live off his savings. In 1949, he moved to the UK to work with Alexander Todd at the University of Cambridge [1,2].

In 1952, he immigrated to Canada to join the research team of Dr. Gordon M. Shrum at the University of British Columbia. In Vancouver, he established an exceptional research program. It was here that Khorana began his pioneering work on the synthesis and structure of nucleic acids, laying the groundwork for his groundbreaking discoveries in deciphering the genetic code [1,2].

In 1960, he joined the University of Wisconsin-Madison, where he focused his research on the genetic code and the chemical synthesis of a transfer RNA gene. His research emphasized understanding the chemical structure of nucleic acids, particularly RNA, and their role in protein synthesis. During his tenure at the University of Wisconsin, he was awarded the Nobel Prize in 1968 [1,2].

In 1970, he transitioned to the Massachusetts Institute of Technology in Cambridge, where he shifted his focus to the study of membranes and signal transduction. He dedicated over three decades to investigating these areas until his retirement in 2007 [1,2].

Deciphering the genetic code

Central to Khorana's research was unraveling the complex biochemical process of gene expression. With a background in chemistry, he applied his knowledge to solve biological problems. In 1959, he utilized carbodiimides (a class of organic compounds) to synthesize both deoxyribonucleotide and ribonucleotide triphosphates, as well as coenzyme A. This work gained international recognition [2,3]. By synthesizing RNA molecules with specific nucleotide sequences and observing the resulting protein products, his team deduced the correspondence between codons in mRNA and amino acids. His work provided crucial insights into how genetic information encoded in DNA and RNA is expressed by being translated into functional proteins [3].

Synthesis of artificial genes

One of Khorana’s most notable achievements occurred in 1970 when he synthesized the first fully functional artificial gene outside of a living organism. By stringing together nucleotides in a precise sequence, Khorana created a synthetic DNA molecule that coded for a specific amino acid sequence. In 1979, he demonstrated that this gene was functional in a bacterium [4]. Khorana's pioneering work laid the foundation for many applications in biotechnology, including the production of recombinant proteins, gene therapy, and the development of genetically modified organisms [4].

Legacy and impact

In recognition of his contributions to science, Khorana received numerous accolades and honors. The 1968 Nobel Prize for Physiology or Medicine was jointly awarded to three scientists: Har Gobind Khorana, Marshall W. Nirenberg (1927-2010), and Robert W. Holley (1922-1993). Khorana contributed to the development of methods for investigating nucleic acid structure and demonstrated how RNA can specify protein structure. Nirenberg devised procedures for decoding the genetic code within living cells, while Holley developed techniques for determining the nucleic acid structure and successfully sequenced the entire nucleotide sequence of alanine transfer ribonucleic acid (tRNA).

He was also awarded the Padma Vibhushan in 1969, India's second-highest civilian award, in recognition of his outstanding achievements. In 1995, Khorana was commemorated on a stamp (Scott No. 2219d) issued by St. Vincent and the Grenadines [2]. Khorana also received the Albert Lasker Basic Medical Research Award in 1968 and the National Medal of Science in 1987 [3]. The University of Wisconsin launched a fellowship initiative aimed at offering young students from India similar opportunities to those once provided to Dr. Khorana [3].

Beyond his scientific achievements, Khorana was known for his humility, integrity, and dedication to education. He was a deep thinker, a brilliant strategist, and excelled as a collaborator and organizer. Throughout his academic career, he published over 450 papers, mentored over 150 postgraduate students, and inspired countless graduate students to pursue careers in science [3].

## Conclusions

Dr. Har Gobind Khorana's contributions to molecular biology and biotechnology have left an enduring legacy that continues to shape the scientific landscape. As we remember his life and achievements, we honor his curiosity and drive to learn, which shaped his incredible career. His life motivates others to keep exploring and discovering new things about the world, inspiring people to ask questions and unravel the mysteries of the universe.
